# Supplementary material for: An antisense RNA regulates production of DnaA and affects sporulation in Bacillus subtilis
Source: PLoS Genet. 2025 May 14;21(5):e1011625. doi: 10.1371/journal.pgen.1011625 (PMC12112137; doi:10.1371/journal.pgen.1011625)
Supplement: S1 Table — List of primers used for RT-qPCR and pPCR, including primers for measuring mRNA from dnaA, dnaN, and gyrA (for normalization) and for determining the ori to ter ratio. (PDF) [file pgen.1011625.s001.pdf]

S1 Table. Primers for RT-qPCR and qPCR[1].

Target[2]	Primer 1	Primer 2
dnaA	CGTCAAATCGCCATGTACTTATC	ATCACGTCCTCCAAACTCTTC
dnaN	CCGATGGCAACTGTAGAAATTG	ATCAGCATCCAGTCCGTTTAG
gyrA	TGGAGCATTACCTTGACCATC	AGCTCTCGCTTCTGCTTTAC
ori	TTGCCGCAGATTGAAGAG	AGGTGGACACTGCAAATAC
*te*r	CGCGCTGACTCTGATATTATG	CAAAGAGGAGCTGCTGTAAC

[1]Primers are indicated in the 5′ to 3′ direction.

[2] Primers for *dnaA*, *dnaN*, and *gyrA* were used in RT-qPCR to measure relative mRNA levels. Primers for *ori* and *ter* were used in qPCR to measure the amount of origin and terminus DNA in cells as an indication of replication.
