# Supplementary material for: An antisense RNA regulates production of DnaA and affects sporulation in Bacillus subtilis
Source: PLoS Genet. 2025 May 14;21(5):e1011625. doi: 10.1371/journal.pgen.1011625 (PMC12112137; doi:10.1371/journal.pgen.1011625)
Supplement: S2 Table — Table includes accession numbers, species, and strain information for genomes that were analyzed for possible promoters and DnaA binding sites between the dnaA promoter and open reading frame, similar to those found in B. subtilis and B. licheniformis. (PDF) [file pgen.1011625.s002.pdf]

S2 Table. Reference genomes.

Accession	Species	Strain	Assembly Level	Date
GCF_001191605.1	*B. altitudinis*	GR-8	Complete Genome	8/3/15
GCF_000242855.2	*B. amyloliquefaciens*	IT-45	Complete Genome	2/13/13
GCF_000008445.1	*B. anthracis*	Ames Ancestor; A2084	Complete Genome	7/9/04
GCF_000742675.1	*B. atrophaeus* (*globigii*)	BSS	Chromosome	8/21/14
GCF_020002245.1	*B. badius*	NBPM-293	Complete Genome	9/15/21
GCF_004124315.2	*B. cabrialesii*	TE3	Complete Genome	5/9/22
GCF_002220285.1	*B. cereus*	FORC_047	Complete Genome	7/17/17
GCF_002250945.2	*B. cytotoxicus*	CH_13	Complete Genome	5/18/18
GCF_007995155.1	*B. dafuensis*	FJAT-25496	Complete Genome	8/14/19
GCF_012955605.1	*B. fonticola*	CS13	Complete Genome	5/3/20
GCF_004103615.1	*B. glycinifermentans*	SRCM103574	Complete Genome	1/23/19
GCF_001278705.1	*B. gobiensis*	FJAT-4402	Chromosome	9/4/15
GCF_004006435.1	*B. halotolerans*	ZB201702	Complete Genome	1/9/19
GCF_014042035.1	*B. haynesii*	P19	Complete Genome	8/2/20
GCF_019598985.1	*B. inaquosorum*	CCSR02	Chromosome	8/15/21
GCF_000473245.1	*B. infantis*	NRRL B-14911	Complete Genome	9/30/13
GCF_002074095.1	*B. licheniformis*	SCDB 14	Complete Genome	3/29/17
GCF_009739945.1	*B. luti*	FJ	Complete Genome	12/11/19
GCF_012648005.1	*B. mojavensis*	UCMB5075	Complete Genome	4/22/20
GCF_018739485.1	*B. mycoides*	BPN36/3	Complete Genome	6/7/21
GCF_018141025.1	*B. nitratireducens*	BM02	Complete Genome	4/27/21
GCF_020861345.1	*B. pacificus*	anQ-h4	Complete Genome	11/14/21
GCF_002993925.1	*B. paralicheniformis*	Bac84	Complete Genome	3/12/18
GCF_000161455.1	*B. pseudomycoides*	DSM 12442	Chromosome	5/29/09
GCF_023238245.1	*B. rugosus*	A78.1	Complete Genome	5/4/22
GCF_008244765.1	*B. safensis*	PgKB20	Complete Genome	9/3/19
GCF_020519665.1	*B. shivajii*	JCM 32183	Complete Genome	10/18/21
GCF_001050115.1	*B. smithii*	DSM 4216	Complete Genome	7/8/15
GCF_012225885.1	*B. tequilensis*	EA-CB0015	Complete Genome	4/9/20
GCF_000161615.1	*B. thuringiensis* (*berliner*)	ATCC 10792	Chromosome	5/29/09
GCF_016605985.1	*B. toyonensis*	P18	Complete Genome	1/11/21
GCF_011040455.1	*B. tropicus*	AOA-CPS1	Chromosome	2/29/20
GCF_003667885.1	*B. vallismortis*	Bac111	Complete Genome	10/21/18
GCF_000769555.1	*B. velezensis*	JS25R	Complete Genome	10/28/14
GCF_001889165.1	*B. weihaiensis*	Alg07	Complete Genome	12/5/16
GCF_008807735.1	*B. wiedmannii*	SR52	Complete Genome	10/3/19
GCF_001857925.1	*B. xiamenensis*	VV3	Complete Genome	10/31/16
