# Supplementary figures and images for: An antisense RNA regulates production of DnaA and affects sporulation in Bacillus subtilis
Source: PLoS Genet. 2025 May 14;21(5):e1011625. doi: 10.1371/journal.pgen.1011625 (PMC12112137; doi:10.1371/journal.pgen.1011625)

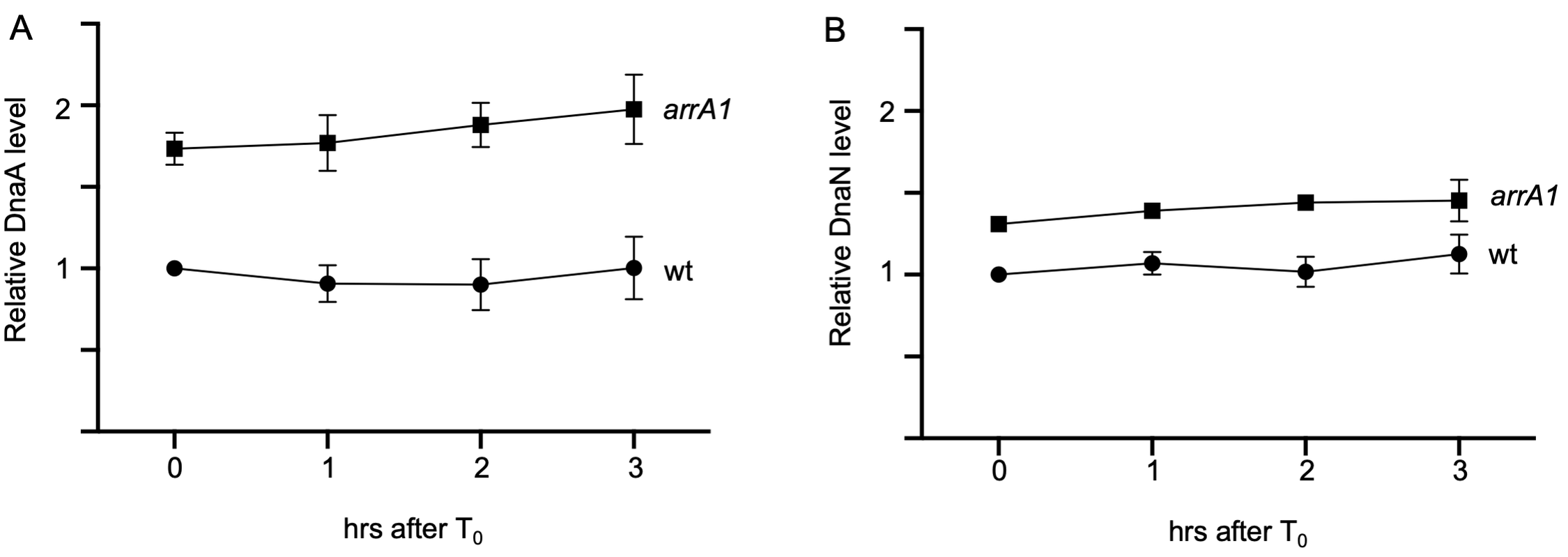

Supplement: S1 Fig — Wild type (AG174) and arrA1 mutant (ELS393) cells were grown in sporulation medium (DSM) and the amount of (A) DnaA and (B) DnaN was measured by western blotting at the times indicated. Time 0 (T0) represents entry into stationary phase. The OD600 of the culture at the time of sampling was used to load equivalent amounts of lysate in each lane. Three independent experiments were performed for both strains and the amount of each protein was normalized to that in wild type cells at T0 for each experiment. The average for each time point ± the standard error of the mean is presented. (TIF) [file pgen.1011625.s003.tif]
