# Supplementary material for: An antisense RNA regulates production of DnaA and affects sporulation in Bacillus subtilis
Source: PLoS Genet. 2025 May 14;21(5):e1011625. doi: 10.1371/journal.pgen.1011625 (PMC12112137; doi:10.1371/journal.pgen.1011625)
Supplement: S1 File — Images. Images of gels and blots used in Figs 4 and 5. S1A. Images of the northern blot (left) and stained gel (right) that were used for Fig 4A and 4B, respectively. S1B. Images of the western blot (top) and stained gel (bottom) that were used for Fig 5B. (PDF) [file pgen.1011625.s005.pdf]

S1A Raw Images. Images of the Northern blot (left) and stained gel (right) that were used for Figure 4A and B.

lane 1. ELS393 (*arrA1*); RNA0023
lane 2. AG174 (wild type); RNA0022
lane 3. AG174 (wild type); RNA0020
lane 4. ELS393 *(arrA1*); RNA0021
lane 5. Markers

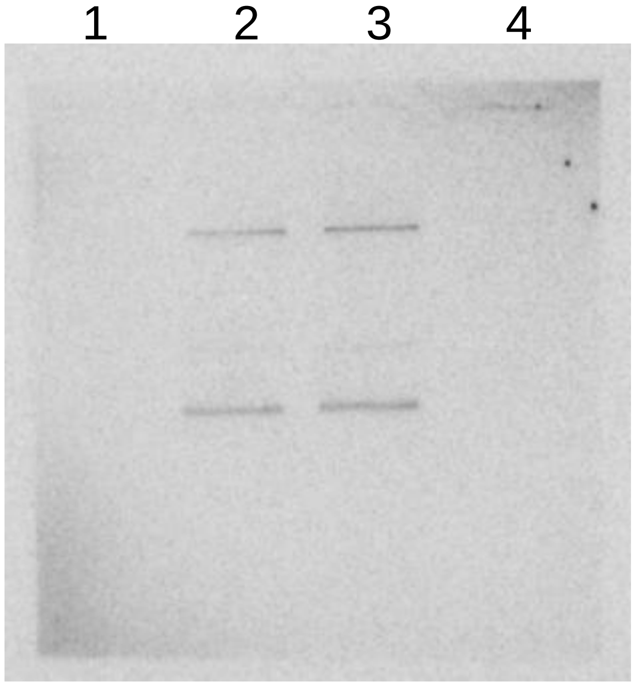

Northern Blot

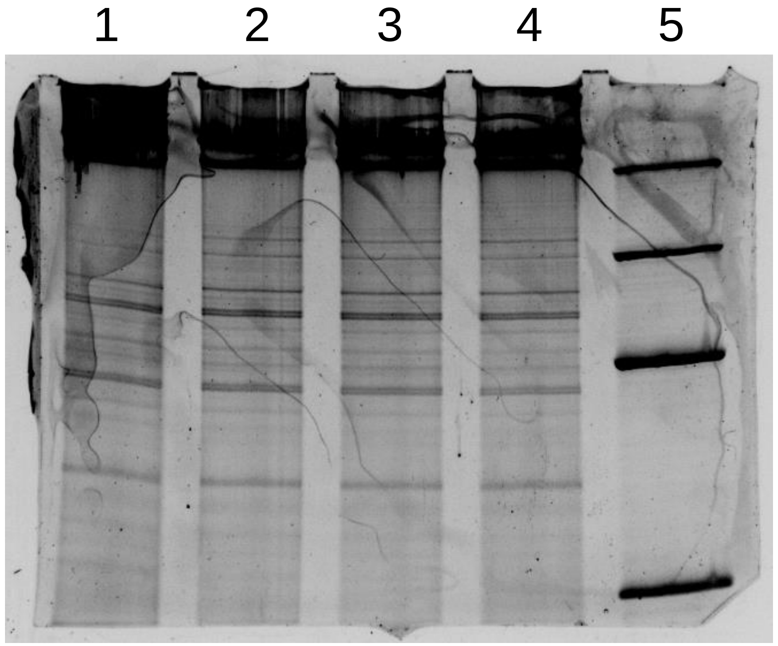

SYBR Gold stain

lanes 3-5 from both images are presented in Fig 4.

S1B Raw Images. Images of the Western blot (top) and stained gel (bpttom) that were used for Figure 5B.

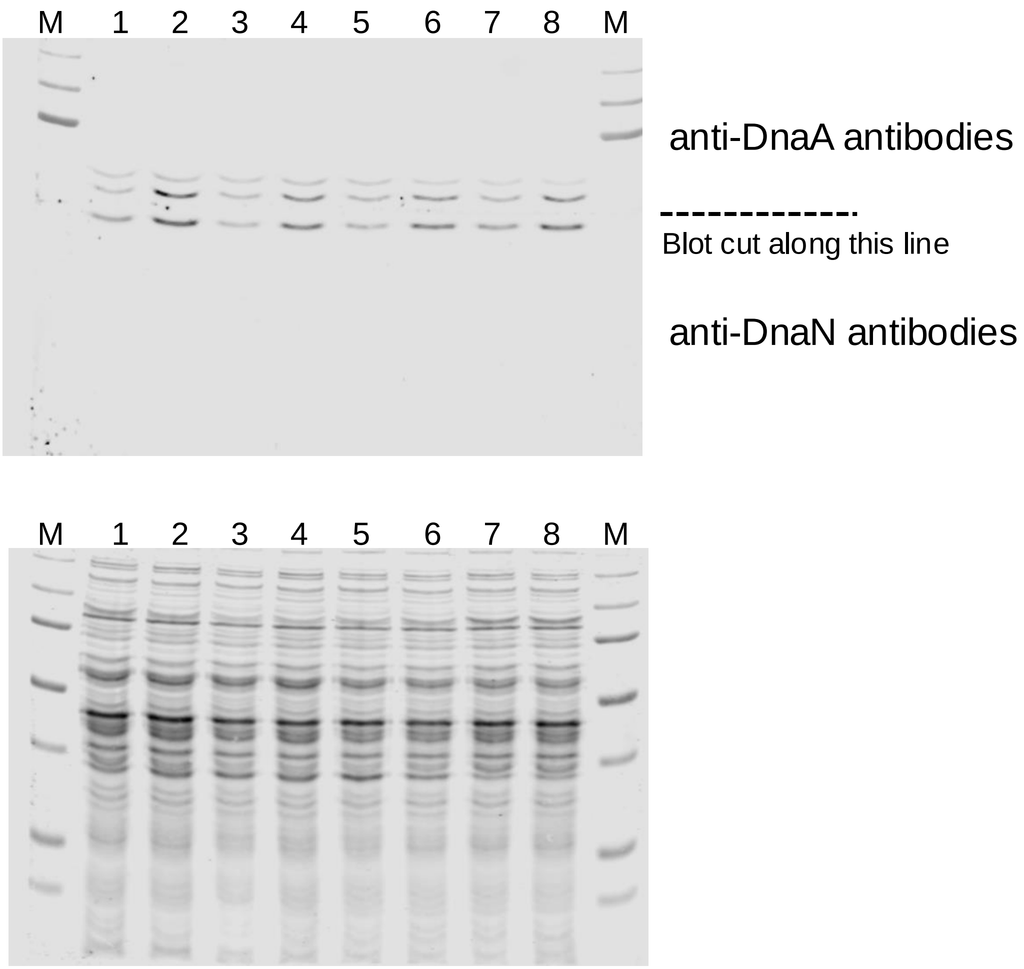

protein stain

M = Markers

lanes 1, 3, 5, 7: Protein from four independent cultures of wild type cells (strain AG174)

lanes 2, 4, 6, 8: Protein from four independent cultures of an *arrA1* mutant (strain ELS393). Each was grown in parallel with the wild type for a given experiment.

The top of the blot was probed with anti-DnaA antibodies. The bottom was probed with anti-DnaN antibodies. Bands were scanned and normalized to the amount of protein in the same lane that was stained for total protein (Methods).
